# Mechanisms, Risk Factors, and Management of Acquired Long QT Syndrome: A Comprehensive Review

**DOI:** 10.1100/2012/212178

**Published:** 2012-04-19

**Authors:** Eleftherios M. Kallergis, Christos A. Goudis, Emmanuel N. Simantirakis, George E. Kochiadakis, Panos E. Vardas

**Affiliations:** Department of Cardiology, University Hospital of Heraklion, 711 10 Heraklion, Crete, Greece

## Abstract

Long QT syndrome is characterized by prolongation of the corrected QT (QTc) interval on the surface electrocardiogram and is associated with precipitation of torsade de pointes (TdP), a polymorphic ventricular tachycardia that may cause sudden death. Acquired long QT syndrome describes pathologic excessive prolongation of the QT interval, upon exposure to an environmental stressor, with reversion back to normal following removal of the stressor. The most common environmental stressor in acquired long QT syndrome is drug therapy. Acquired long QT syndrome is an important issue for clinicians and a significant public health problem concerning the large number of drugs with this adverse effect with a potentially fatal outcome, the large number of patients exposed to these drugs, and our inability to predict the risk for a given individual. In this paper, we focus on mechanisms underlying QT prolongation, risk factors for torsades de pointes and describe the short- and long-term treatment of acquired long QT syndrome.

## 1. Introduction

**Figure 1:**
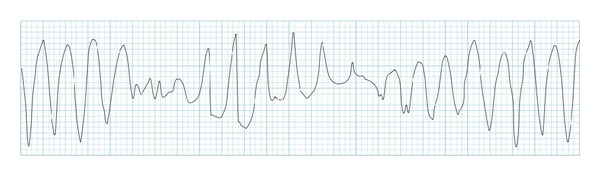
Torsades de pointes.

Acquired long QT syndrome is a disorder of cardiac repolarization most often due to specific drugs, hypokalemia, or hypomagnesemia that may precipitate torsade de pointes and cause sudden cardiac death. Selzer and Wray first reported QT prolongation and ventricular fibrillation as a response to quinidine in 1964 [1]. Two years later, Dessertenne [2] described torsades de pointes, a polymorphic ventricular tachycardia where QRS complexes twist around an isoelectric line in a sinusoidal fashion in an elderly woman with complete atrioventricular block and syncopal attacks (Figure 1). Torsade de pointes is usually self-limited but may degenerate into ventricular fibrillation. The incidence of acquired long QT syndrome is difficult to be estimated. Although the chances of provoking torsades de pointes by a noncardiac medication are generally lower than antiarrhythmic medications, a number of noncardiovascular drugs have been recently withdrawn from market because of unexpected sudden cardiac deaths associated with prolongation of QT interval and torsades de pointes [3]. The frequency of drug-induced long QT syndrome and our inability to predict the risk for a given individual, makes long QT syndrome an important issue for clinicians. This paper focuses on mechanisms underlying QT prolongation, risk factors for torsades de pointes and describes the short- and long-term treatment of acquired long QT syndrome.

## 2. QT Interval Measurement

QT interval on the surface electrocardiogram describes the manifestation of ventricular depolarization and repolarization. It is measured from the beginning of QRS complex to T wave termination and averaged over 3 to 5 beats in a single lead. Longest QT intervals are usually measured in precordial leads and V3 or V4 leads appear more reliable for assessing QT prolongation [4]. Prominent U waves should be included in the measurement if they merge into the T wave. QT interval is influenced by heart rate. The RR interval preceding the QT interval should be measured for rate correction. Several formulae have been proposed for heart rate correction of the QT interval. The most commonly used formulae are Fridericia's cube root formula (QTc = QT/RR1/3) and Bazett's square root formula (QTc = QT/RR1/2). Although there is no consensus on best QTc method, Bazett's formula is considered the gold standard, even though it may overestimate QT prolongation [5]. In general, QT prolongation is considered when the QTc interval is greater than 440 ms, but arrhythmias are most often associated with values of 500 ms or more. QTc interval is longer in adult women because of a relative shortening of the QTc interval in men during adolescence [6]. Intervals of 440 to 460 milliseconds in men and 440 to 470 milliseconds in women are considered borderline [7]. QT intervals may also vary due to ECG acquisition technique, electrolyte imbalance, sympathovagal activity, intra- and interobserver variability, and diurnal variation which can be up to 75–100 ms [8, 9]. It is important to notice that for every individual there is a different relation between the QT interval and the heart rate, and even though rate-correction formulae are useful clinically, they may not be accurate enough, especially when assessing the minor changes of the QT interval induced by drugs.

## 3. Mechanisms of Drug-Induced QT Prolongation

QT interval on the surface electrocardiogram represents the summation of action potentials in ventricular myocytes. QT prolongation entails action potential prolongation, that results from an increase in inward current (e.g., through sodium or calcium channels) or a decrease in outward current (e.g., through potassium channels). Myocardial repolarization is primarily mediated by efflux of potassium ions. Two subtypes of the delayed rectifier potassium current, IKr (rapid) and IKs (slow), are predominantly responsible for repolarization. The two currents have different activation, inactivation, and deactivation characteristics, different sensitivities to blocking drugs [10–12], different rate, and catecholamine sensitivity [13, 14] and were later found to be the result of expression of different genes [15, 16].

The hallmark mechanism of acquired LQTS and TdP is the blockade of IKr by specific drugs [17]. IKr current proteins are encoded by the human ether-a-go-go-related gene HERG (now termed KCNH2) [18]. Two structural characteristics account for the unusual susceptibility of IKr channels to various drugs [19]. First, the presence of aromatic aminoacids (Tyr652 and Phe656) with side chains directed towards the large central cavity of the pore region provides high-affinity binding sites for many of compounds. These amino acids are not present in most other potassium channels, and mutation of KCNH2 at these sites to other amino acids reduces binding affinity of several drugs. Second, while most potassium channels contain two proline residues in the helix that forms part of the pore that restrict access to the drug binding site, these two prolines are absent in KCNH2. Mutation of these residues to the conserved Pro-Val-Pro results in reduced drug binding [20].

**Figure 2:**
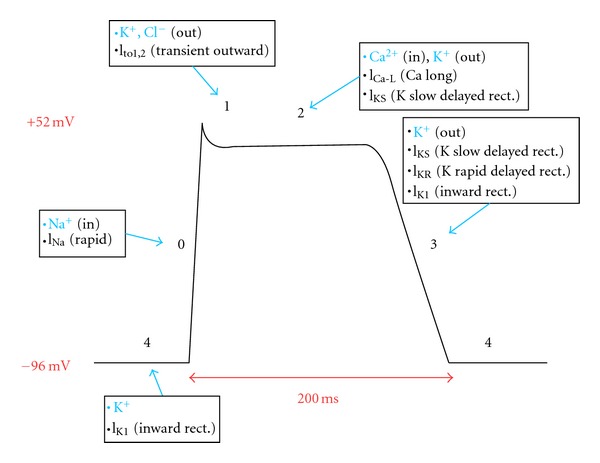
Myocardial action potential. Phase 0 rapid depolarization is mediated by sodium entry into cells. Phase 1 and 3 repolarization results from potassium efflux from cells. Balanced slow calcium entry and potassium exit cause the plateau in phase 2. Potassium reenters and sodium exits cells during phase 4 recovery.

**Figure 3:**
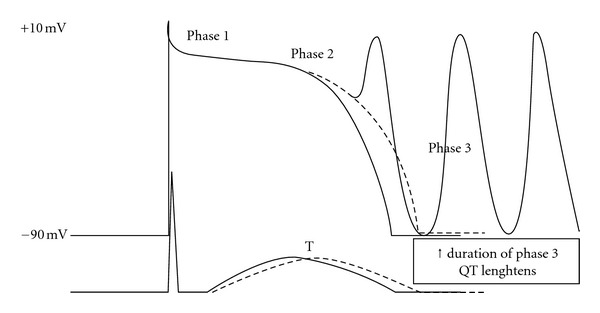
Multiple early afterdepolarizations (EADs) from progressively more negative transmembrane potential.

**Figure 4:**
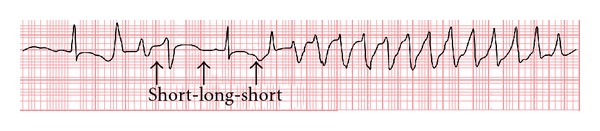
Short-long-short sequence preceding TdP.

Ikr blockade causes a delay in phase 3 rapid repolarization of the action potential (Figure 2), which is reflected by QT prolongation. Prolonged repolarization can cause early afterdepolarizations (EADs) due to activation of inward depolarizing currents (L-type calcium channels or sodium-calcium exchange current) [21], that appear as depolarizing oscillations in membrane voltage during phases 2 and 3 of the action potential (Figure 3). EADs that reach threshold voltage can cause a ventricular extrasystole preceded by a long QT interval on the surface ECG. On the other hand, dispersion of refractoriness due to heterogeneity in ventricular repolarization can create zones of unidirectional block. Repetitive extrasystoles, unidirectional block and zones of slow conduction can lead to reentry and TdP [22]. Torsades de pointes is usually preceded by a short-long-short ECG sequence (Figure 4) [23]. In this case one or more premature ventricular complexes are followed by a compensatory pause. The subsequent sinus beat may have an especially long QT and deformities of T or U waves. This sinus beat is followed by another premature ventricular complex that precipitates torsades de pointes [24].

Several other ECG variables besides QT interval have been proposed to be predictors of TdP. QT dispersion, which represents the difference between maximum and minimum QT intervals, was supposed to be a more direct measure of spatial heterogeneity of repolarization [25], but proved to be a disappointing tool, because it is mostly dependent on T wave morphology [26]. An increasing number of basic and clinical studies suggest that the interval from the peak to the end of the electrocardiographic T wave (Tp-e) corresponds to transmural dispersion of repolarization [27–29]. Prolonged QTc interval and Tpeak-Tend was found to correlate with increased risk for torsades de pointes during acquired bradyarrhythmias [30]. The Tp-e/QT ratio serves as a more sensitive index of arrhythmogenesis as it provides an estimate of dispersion of repolarization relative to total duration of repolarization. Thereby, it eliminates the confounding effects of variability of heart rate and interindividual variation of QT interval [31]. Outlined evidence clearly suggests the applicability of Tp-e/QT ratio as a potentially important index of arrhythmogenesis, even though direct validation of Tp-e interval as a body surface index of transmural dispersion of repolarization is still lacking [32]. More recent studies have also provided guidelines for the estimation of transmural dispersion of repolarization in the case of more complex T waves, including negative, biphasic, and triphasic T waves [33]. In such cases, the interval from the nadir of the first component of the T wave to the end of the T wave was shown to provide an electrocardiographic approximation of transmural dispersion of repolarization. T-wave alternans or a change in amplitude or polarity of the T-wave on alternating beats has been observed in LQTS as a precursor to TdP [34]. T-wave alternans is thought to result from alternation of the M-cell APD, leading to exaggeration of transmural dispersion of repolarization during alternate beats, and thus the potential for development of TdP [35].

Abnormal, giant T-U waves and a slow QRS upstroke separate TdP initiation in LQTS patients from PVCs in other heart disease and from other PVCs in LQTS patients. Abnormal T-U waves support the notion that EADs are the trigger for TdP in LQTS. If found, they may be an indicator for imminent risk of TdP [36]. Short-term variability of QT intervals (as measured from 30 consecutive beats) is increased in patients with a history of drug-induced long QT syndrome, suggesting that it could prove to be a useful noninvasive, easily obtainable parameter aiding the identification of the patient at risk for potentially life-threatening arrhythmia in the context of drugs with QT prolonging potential [37].

Although evaluating the effect of a new drug on the QTc interval is important, conclusions on the potential clinical risk of TdP associated with its use, based solely on its ability to prolong the QTc interval, might turn out to be highly flawed. Tpeak-Tend measurement and Tp-e/QT ratio, giant T-U waves, slow QRS upstroke, and short-term variability of QT intervals tend to be useful clinical variables to predict risk of TdP.

## 4. Risk Factors

Multiple clinical risk factors (Table 1) are often present in an individual case. These factors provide a starting point for basic research into underlying mechanisms at the genetic, molecular and cellular level. The occurrence of drug-induced LQTS is unpredictable in any given individual, but a common observation is that most patients have at least one identifiable risk factor in addition to drug exposure [38].

A female preponderance has been consistently observed in multiple studies, with TdP occurring two to three times more commonly in women than in men [39]. These clinical observations, coupled with the finding that the QT shortens after puberty in males but not female [40], suggest that sex hormones modulate repolarization. Testosterone, by increasing IKr, shortens QTc and has been implicated as the major factor lowering risk of TdP in males [41].

Hypokalemia is another common risk factor in drug-induced LQTS. Low extracellular potassium paradoxically reduces IKr by enhanced inactivation [42] or exaggerated competitive block by sodium [43]. As a result, hypokalemia prolongs the QT interval. However, the fact that low extracellular potassium increases IKr blockade by drug, is of most importance in clinical practice [44]. Correction of extracellular potassium to the high normal range can shorten QT interval and associated morphological abnormalities [45, 46].

Pauses, usually after an ectopic beat, precipitate drug-induced TdP. It is presumed that pause generates the dispersion of many electrophysiological properties, notably repolarization times, that underlie torsades de pointes [47]. In Holter recordings of patients with drug-induced TdP an increase in underlying sinus heart rate was reported in the minutes prior to an event [48]. This finding suggests that a pause in the setting of heightened sympathetic activation and long QT intervals may be especially arrhythmogenic.

The period shortly after conversion of atrial fibrillation is characterized by increased risk of torsades de pointes. Studies using QT/RR plots during atrial fibrillation have shown rate-independent QT prolongation after conversion to sinus rhythm [49]. Dofetilide causes only minor QT prolongation during atrial fibrillation, but significantly more QT prolongation when given to the same patients after cardioversion to sinus rhythm [50]. Congestive heart failure [51] and left ventricular hypertrophy are other high-risk situations for drug-induced torsades de pointes, but further investigation is needed on molecular and cellular mechanisms.

For the majority of drugs (with the exception of class IA drugs), risk increases with higher drug concentrations. Class IA drugs (quinidine, disopyramide, and procainamide) block outward potassium currents and inward sodium currents. Sodium current blockade increases as serum levels increase, but potassium current blockade predominates at low serum levels. Therefore, TdP frequently occurs at low or subtherapeutic serum levels [52]. Administration of more than one drug that prolong repolarization increases the risk of drug-induced LQTS, but in most cases the mechanism of increased risk is due to drug-drug interactions altering metabolism, rather than simple additive effects on IKr. Cytochrome P450 superfamily of proteins is responsible for the metabolism of most of the drugs by liver and CYP3A4 is the predominant cytochrome P450. Coadministration of drugs which are substrates for CYP3A4 and/or IKr blockers results in further QT prolongation. Drugs that prolong QT interval and inhibitors of CYP3A4 are shown in Tables 2 and 3. Amiodarone rarely causes torsades de pointes despite significant QT prolongation. Amiodarone blocks IKr without reverse use dependence and prolongs action potential duration in a homogenous manner, thus reducing heterogeneity of refractoriness and making myocardium less susceptible to reentry. Additional electrophysiologic effects that explain its safety include noncompetitive *β* antagonism and inward L-calcium channel blockade which may reduce EADs [53]. The incidence of torsades de pointes at currently used doses is <1% [54, 55] while with sotalol the incidence ranges from 0.8 to 3.8% [56] and with ibutilide from 3.6 to 8.3% [57, 58]. Verapamil, even though a potent IKr blocker, rarely causes torsades de pointes [59]. Verapamil reduces EADs by blocking inward calcium current [60], reduces transmural dispersion of refractoriness, and shortens QT interval and the incidence of TdP in a model of acquired LQTS from combined IKs and IKr block [61].

Subclinical mutations or polymorphisms in congenital LQTS genes have been described as a risk factor for the drug-associated form [38]. Patients with subclinical congenital LQTS may develop TdP after exposure to a QT prolonging agent [62]. In approximately hundred patients with drug-induced form of LQTS, congenital LQTS disease genes were identified in a percentage of 5–10% and their mutations classified them as having the congenital syndrome [63]. Identification of these cases emphasizes the increasing recognition of incomplete penetrance. As a result, many patients with the congenital syndrome have normal baseline ECGs, but they may be at increased risk for torsades during drug challenge [64, 65]. First-degree relatives of patients with drug-induced TdP exhibit greater abnormalities of cardiac repolarization in comparison with first-degree relatives of patients tolerating QT-prolonging antiarrhythmic therapy [66].

This variability in the risk of torsade de pointes involves defining the molecular mechanisms that control the duration of action potential and the QT interval in the normal heart and in diseases such as the congenital long QT syndrome or heart failure. The term repolarization reserve has been proposed as a unifying framework for analysis of risk factors and their clinical mechanisms [67]. Repolarization reserve characterizes the capacity of the myocardium to affect orderly and provide rapid repolarization through normal mechanisms. In normal hearts, repolarization is accomplished by multiple and redundant mechanisms. The presence of a single risk factor is usually insufficient to elicit a LQTS phenotype. Multiple subclinical lesions are necessary in the repolarization process before superimposition of an IKr-blocking drug can produce marked action potential prolongation and torsades de pointes.

Genetic modulation of repolarization reserve is thought to contribute to interindividual differences in susceptibility to QT-prolonging drugs. Genetic variations in *KCNH2*, *KCNE*, *KCNE2*, and *ANKB *have been identified in some patients with drug-induced torsades de pointes [68, 69]. The gene in which mutations seem most common is KCNQ1, whose expression results in IKs [62, 63]. Studies in human myocytes and in computational models have implicated variability in IKs amplitude as a major contributor to variability in response to IKr block (i.e., to repolarization reserve) [70, 71]. IKs amplitude is readily increased by interventions such as adrenergic stimulation [72] or endothelin [73], but also by compensatory increase through posttranscriptional upregulation of underlying units of IKs, likely mediated by microRNA changes due to sustained reductions of IKr [74]. The way in which the above-mentioned and other mechanisms might contribute to IKs regulation during challenge with an IKr blocker remains a broad area for investigation, both at the clinical level and at the molecular level.

Current studies investigate the relationship of common variants within the human genome termed polymorphisms and variable risk for torsades de pointes. The most compelling example to date is a single nucleotide polymorphism (SNP), common in African Americans, that results in substitution of a tyrosine for serine at position 1103 of the cardiac sodium channel. The *SCN5A *variant S1103Y points to the potential role of ethnicity as a genetic determinant of repolarization reserve [75]. This is further confirmed by the unique distribution of certain ion channel variants across different ethnic groups [76–78]. Pharmacogenetics additionally may determine arrhythmia risk in patients with acquired LQTS. Genetically determined reduced activity of cytochrome P450 enzyme CYP3A4 may decrease efficient metabolism of the QT prolonging drugs thioridazine, erythromycin, and terfenadine [79].

## 5. Treatment

The cornerstone of the management of acquired LQTS includes the identification and discontinuation of any precipitating drug and the aggressive correction of any metabolic abnormalities, such as hypokalemia or hypomagnesemia. Most of the episodes of torsade de pointes are short-lived and terminate spontaneously. However, prolonged episodes result in hemodynamic compromise and require immediate cardioversion.

Short-term treatment of the syndrome focuses on prevention of recurrence of torsade de pointes and includes administration of intravenous magnesium sulfate and temporary transvenous cardiac pacing. Intravenous isoproterenol is rarely needed. Important step in the management of acquired LQTS is withdrawal of offending agents and correction of electrolyte abnormalities [80]. The effectiveness of lidocaine, phenytoin, or atropine even though reported to be beneficial is uncertain [81].

Intravenous magnesium is the agent of choice for immediate treatment of torsade de pointes irrespective of the serum magnesium level. 2 g bolus of magnesium sulfate is followed by intravenous infusion of magnesium at a rate of 2–4 mg per minute [82]. The mechanism by which magnesium prevents the recurrences of torsade de points is unclear. Its action is probably mediated through blockage of sodium or calcium currents. The only side effect of intravenous magnesium is flushing during the bolus injection. Administration of potassium is an important adjunct to intravenous magnesium for the short-term prevention of torsade de pointes, especially if the serum potassium level is low. Serum potassium should be maintained in the high normal range. Overdrive transvenous pacing shortens QTc and is highly effective in preventing recurrences of torsades de pointes [83], especially when they are precipitated by a pause or bradycardia. Short-term pacing rates of 90 to 110 beats/min are recommended. Cardiac pacing prevents pauses and shortens the QTc interval by enhancing the repolarizing potassium currents [84]. Isoproterenol is useful if temporary pacing is unavailable or while preparing for transvenous catheter insertion [85]. Unlike acquired LQTS, isoproterenol is contraindicated in patients with congenital LQTS or ischemic heart disease. Side effects include palpitations and flushes.

Long-term treatment is rarely required. Conditions that predispose to electrolyte imbalance must be corrected. In cases of sick sinus syndrome or atrioventricular block and bradycardia, permanent pacing may be indicated [86].

## Figures and Tables

**Table 1:** Risk factors for drug-induced torsade de pointes.

Female sex
Hypokalemia
Bradycardia
Recent conversion from atrial fibrillation
Congestive heart failure
Left ventricular hypertrophy
High drug concentrations
Rapid rate of intravenous infusion with a QT-prolonging drug
Base-line QT prolongation
Subclinical long QT syndrome
Ion-channel polymorphisms
Severe hypomagnesemia

**Table 2:** Drugs implicated in torsades de pointes.

(1) Antiarrhythmic medications
Class IA (Quinidine, Procainamide, and Disopyramide)
Class III (Dofetilide, Ibutilide, Sotalol, and Amiodarone)
Class IV (Verapamil)
(2) Promotility medications
Cisapride
(3) Antimicrobial medications
Macrolides
Erythromycin
Clarithromycin
Fluoroquinolones
Antiprotozoals
Pentamidine
Antimalarials
Chloroquine
(4) Antipsychotic medications
Phenothiazine neuroleptics
Thioridazine
Chlorpromazine
Butyrophenone neuroleptics
Haloperidol
(5) Miscellaneous medications
Arsenic trioxide
Methadone

**Table 3:** Inhibitors of CYP3A4.

(1) Antihypertensive medications
Dihydralazine
Diltiazem
Verapamil
(2) Antidepressant and anxiolytic medications
Fluoxetine
Midazolam
(3) Antimicrobial medications
Macrolides
Clarithromycin
Erythromycin
Isoniazid
HIV agents
(4) Endocrine medications
Contraceptives
Ethinylestradiol
Antiprogesterone agent
Estrogen receptor modulators
Tamoxifen
(5) Food and herbal constituents
Bergamottin (grapefruit juice)
Glabridin (licorice)
